# Coping strategies among emerging adult children of parents with a mental illness

**DOI:** 10.3389/fpsyg.2026.1760621

**Published:** 2026-04-15

**Authors:** Franziska Sawitzki, Nicola Großheinrich

**Affiliations:** Department of Social Sciences, Catholic University of Applied Sciences North Rhine-Westphalia (katho), Cologne, Germany

**Keywords:** coping strategies, emerging adult children of parents with a mental illness, mental health, parental mental illness, transgenerational transmission of psychopathology

## Abstract

Coping is central to the transgenerational transmission of psychopathology, yet little is known about coping strategies used by emerging adult offspring of parents with mental illness or their links to mental health. This cross-sectional online study, conducted in Germany, compared coping in 18–30-year-olds with versus without parental mental illness (*N* = 139). Offspring of parents with mental illness reported more *behavioral disengagement* (partial η^2^ = 0.03, *p* = 0.036) and less *positive reframing* (partial η^2^ = 0.04, *p* = 0.027). Greater *substance use* (*b* = 4.50, *p* = 0.031) and *self-blame* (*b* = 7.77, *p* = 0.001) were associated with more psychopathology symptoms, and greater *substance use* (*b* = −0.21, *p* = 0.027) and *self-blame* (*b* = −0.28, *p* = 0.008) with poorer positive mental health. Findings highlight the important role of coping and its links to mental health in emerging adults of parents with mental illness and point to areas which could be targeted by interventions.

## 1 Introduction

The effects of parental mental illness on offspring are well-documented across childhood and adolescence (Mattejat and Remschmidt, 2008; van Santvoort et al., 2015), yet the transition from adolescence to adulthood in these individuals often remains underexplored. Emerging adulthood (approximately ages 18–30) is characterized by identity exploration, instability, self-focus, feeling “in-between,” and optimism about the future (Arnett, 2014; Arnett et al., 2014). Although young people gain independence during this period, they typically remain attached to parents and continue to seek support (Lippold et al., 2024). This developmental window is a high-risk period for the onset of mental and substance use disorders, with roughly three-quarters of lifetime cases emerging by age 24 (Adams et al., 2013; Kessler et al., 2005). Despite the risk of chronicity and downstream physical health consequences (Adams et al., 2013), mental health service utilization among emerging adults remains low (Andrews et al., 2001).

For emerging adult children of parents with a mental illness (COPMI), the developmental tasks unfold in the context of enduring impacts of growing up with parental psychopathology which confer additional risk (Steele and McKinney, 2020). Identifying risk and protective processes is essential to prevent disorder onset and to interrupt the transgenerational transmission of psychopathology.

In Hosman et al.’s (2009) developmental model of the transgenerational transmission of psychopathology, coping - alongside genetic, social and individual factors - is a central mechanism. Coping can be defined as “different ways in which people respond to stress” (Carver et al., 1989, p. 267). Whereas many studies examine coping among children and adolescents of parents with a mental illness (e.g., Anderson et al., 2023; Vreeland et al., 2019; Wong and Power, 2023), fewer focus on emerging adults. In a recent systematic review of qualitative studies, only 28.6% of included studies addressed emerging adults (Sawitzki et al., 2025). This gap is worth mentioning as coping repertoires are more differentiated and flexible in emerging adulthood, affording new possibilities for adaptive regulation (Skinner and Zimmer-Gembeck, 2016).

Recent work underscores the influence of parental modeled coping and family climate on offspring coping (Brady et al., 2024; Gervais and Jose, 2024; Liga et al., 2020; Stavish and Lengua, 2023). In families affected by parental mental illness, the socialization pathways may be altered or disrupted: Given potentially harsher family environments and reduced parental availability, offspring of parents with mental illness may both require and engage in greater emotional and instrumental support. Consistent with this, emerging adults exposed to parental mental illness report significantly higher support-seeking than controls without such exposure (Goodday et al., 2019; Mitchell and Abraham, 2018). At the same time, anxiety or depressive symptoms in parents may model avoidance-based coping that is transmitted to offspring and confers risk; correspondingly, disengagement coping is positively associated with psychopathology among children and adolescents in these families (Gruhn et al., 2019; van der Sanden et al., 2016; Wong and Power, 2023).

To identify coping strategies as potential risk or protective factors among emerging adult children of parents with a mental illness, the present study examined: the extent to which these emerging adults differ from peers without such experience in their use of coping strategies; and the associations between these coping strategies and health status within this elevated-risk group. We investigate whether emerging adult children of parents with a mental illness will report significantly greater use of support-seeking coping strategies (Brief COPE emotional support and instrumental support subscales) and greater use of avoidant coping strategies (Brief COPE self-distraction, denial, and behavioral disengagement subscales) than peers without a parent with a mental illness (Goodday et al., 2019; Mitchell and Abraham, 2018). The secondary aim was to test, among emerging adult children of parents with a mental illness, whether greater avoidant coping (Brief COPE self-distraction, denial, and behavioral disengagement subscales) will be positively associated with psychological distress and negatively associated with positive mental health (Wong and Power, 2023).

## 2 Method

### 2.1 Sample

Eligible participants for this study included both individuals who grew up with a parent with a mental illness and those who did not report a parental mental disorder. Participants were required to be between 18 and 30 years old and fluent in German. The parental mental illness was assessed by the participants; diagnoses could be specified if known. The final sample comprised 139 participants aged 18–30 years (*M* = 22.6, *SD* = 2.89). Among them, 50 participants were emerging adults with a parent with a mental illness (age range 19–30, *M* = 22.40, *SD* = 2.72), and 89 were from the comparison group (age range 18–30, *M* = 22.64, *SD* = 2.99).

Demographic characteristics of both groups are shown in Supplementary Table S1.

An *a priori* power analysis was conducted using G*Power version 3.1.9.7 (Faul et al., 2007) to determine the minimum sample size needed to test the study hypotheses. To test the first research question, the results indicated that a sample size of *N* = 136 would be required to achieve 80% power for detecting a medium effect size (*f^2^* = 0.15) at a significance level of *α* = 0.05 for the MANOVA (Global effects). For the linear regression analyses, the required sample size was *N* = 135, for a number of 14 independent variables, a power of 80%, a medium effect size (*f^2^*) of 0.15 and a significance level of α = 0.05. Therefore, we assumed a minimum sample size of 136 participants to be serving our needs.

Participant-reported characteristics of their parents’ mental illness and background information can be found in Supplementary Table S2.

### 2.2 Procedure

Participants were recruited through word of mouth, social media posts, and advertisements in university classes for Bachelor students in social work. Recruitment was conducted at local university in a metropolitan area of Germany, where ethical approval was obtained. Participants were given general information about the study and a link or QR-code to access the survey. Additionally, the processing time of participants was screened, in case of a processing time less than 5 min, datasets were excluded. Data collection was carried out from December 2023 to August 2024. Before participating, individuals read an explanatory statement and provided informed consent. The online survey took approximately 20 min to complete. Participation was voluntary and anonymous, and participants were informed that they could withdraw at any time without any consequences. As an incentive, three 50 Euro vouchers for various stores were offered.

### 2.3 Questionnaire

The online survey included assessments of coping strategies using the Brief COPE (Carver, 1997), and of mental health status using the Brief Symptom Inventory-18 (Derogatis, 2000) and the Positive Mental Health Scale (Lukat et al., 2016). Additionally, demographic data were collected, along with assessments of parental mental illness. Participants were asked to indicate whether they had grown up with a parent affected by mental illness, and, if possible, the diagnosis of the affected parent. Subjective social status was assessed by using a 10-point scale (Hoebel et al., 2013), where higher values indicated a higher perceived status in society.

#### 2.3.1 Coping

The Brief COPE is a shortened version of the COPE inventory (Carver et al., 1989), comprising 28 items that measure 14 distinct coping strategies (Carver, 1997). These 14 two-item scales include active coping, planning, positive reframing, acceptance, humor, religion, emotional support, instrumental support, self-distraction, denial, venting, substance use, behavioral disengagement, and self-blame. Given that parental mental illness constitutes a significant stressor with potential to influence all coping strategies, we employed all 14 coping scales to capture a comprehensive profile of coping strategies. Participants were instructed to reflect on challenging childhood experiences (including those specifically related to parental mental illness for affected emerging adults) and to indicate their coping strategies for managing these situations. Responses were recorded on a Likert scale from 0 (not at all) to 3 (very much). Higher means indicated greater use of the respective coping strategy. Cronbach’s alpha values showed strong internal consistency for almost all scales. The three scales planning (COPMI *α* = 0.36; comparison group α = 0.45; total sample α = 0.42), self-distraction (COPMI α = 0.42; comparison group α = 0.32; total sample α = 0.32) and venting (COPMI α = 0.64; comparison group α = 0.47; total sample α = 0.54) exhibited inadequate internal consistency. These scales, using a 0.60 Cronbach-alpha cut-off, were excluded from all further analyses. The remaining COPE subscales showed Cronbach-alpha values from 0.64 (subscale self-blame in the comparison group) to 0.93 (subscale substance use for COPMI) and were consistent across groups. The study utilized the German version of the Brief COPE (Carver, 2022).

#### 2.3.2 Mental health

In the current study, the mental health status of the emerging adult child was assessed. Participants were asked to evaluate their own mental health, using two instruments: the Mini-SCL (Franke, 2017) and the Positive Mental Health Scale (Lukat et al., 2016).

The Mini-SCL (Franke, 2017), also known as the Brief Symptom Inventory-18 (BSI-18; Derogatis, 2000), is a shortened version of the Brief Symptom Inventory (Franke, 2000). Both inventories are based on the widely used Symptom Checklist (SCL-90; Franke, 2002). The BSI-18 assesses mental stress across three scales: depression, anxiety, and somatization, each consisting of six items. These three scales can be combined into a total score, known as the global severity index. Respondents indicated the extent to which they experienced various symptoms over the past seven days on a Likert scale, from 0 (not at all) to 4 (very much). A sum score was calculated for each scale, with possible scores ranging from 0 to 24. The global score was obtained by summing all three scale scores, resulting in a range between 0 and 72. Internal consistency was strong for each scale, with values ranging from 0.75 for the somatization scale (comparison group) to 0.92 for the aggregated global score (total sample). Cronbach-alpha values were consistent across groups.

Based on the Dual-Factor Model of Mental Health, which encompasses both negative indicators (i.e., psychopathology) and positive indicators (i.e., subjective well-being), the Positive Mental Health Scale (PMH-Scale; Lukat et al., 2016) was employed. This unidimensional scale, comprising nine items, measures positive mental health. Participants rated their agreement with each statement on a Likert scale from 0 (I do not agree) to 3 (I agree). A mean score was calculated, with higher scores indicating better mental health status. Cronbach’s alpha values for the scale were 0.91 for the total sample, 0.91 for the group of COPMI, and 0.90 for the comparison group.

### 2.4 Statistical analyses

IBM SPSS Statistics, version 29, was used for all statistical analyses. Following data preparation, which included inverting items and creating scales, and after testing the statistical requirements, both descriptive and main analyses were conducted. To test the potential differences in coping strategies between individuals with parents with a mental illness and those in the comparison group, a one-way MANOVA was performed. This analysis used the 11 scales with adequate internal consistency of the Brief COPE as dependent variables and was followed by post-hoc ANOVAs. Additional analyses were performed to test the potential impact of sex and emerging adult’s mental health status.

To test the associations between coping strategies and mental health outcomes, we first conducted bivariate correlation analyses. This was followed by two multiple linear regression analyses using the 11 subscales of the Brief COPE (Carver, 2022) as independent variables. The Global Severity Index (GSI) of the Mini-SCL (Franke, 2017) and the Positive Mental Health Scale (Lukat et al., 2016) served as dependent variables.

## 3 Results

### 3.1 Descriptive statistics

The group of young adult children of parents with a mental illness COPMI did not differ significantly from the group of young adults without parents with a mental illness on age, socioeconomic status, educational attainment, employment status, religion, nationality, or migration background. However, a significant difference between both groups on sex distribution was found, with participants who experienced parental mental illness encompassing significantly more female participants. There tend to be more female than male young carers, which may help explain this imbalance (Becker and Sempik, 2019).

Descriptive statistics for mental health status were calculated for both subsamples (see Supplementary Table S3). Emerging adults with a parent with a mental illness showed significantly more symptoms of depression (*MRank* = 82.97) compared to the comparison group (*MRank* = 62.71), *U* = 1576.50, *Z* = −2.86, *p* = 0.004. Similarly, they exhibited significantly more symptoms of anxiety (*MRank* = 85.17) than the comparison group (*MRank* = 61.48), *U* = 1466.50, *Z* = −3.34, *p* < 0.001, and had a higher global severity index (*MRank* = 85.60) compared to those not affected by parental mental illness (*MRank* = 61.24), *U* = 455.00, *Z* = −3.43, *p* < 0.001. No significant differences were found on the somatization scale. Sum scores from the Mini-SCL were standardized, revealing that offspring of parents with a mental illness had a significantly increased GSI, with a standardized value of 66 (95% CI [60, 72]), compared to a norm group. Values starting from 60 indicate an increased to greatly increased symptom burden.

Regarding positive mental health status, the elevated-risk group had significantly worse outcomes (*MRank* = 57.00) than the comparison group (*MRank* = 77.30), *U* = 1575.00, *Z* = −2.86, *p* = 0.004.

### 3.2 MANOVA

To test mean differences in coping strategies, especially in emotional and instrumental support-seeking levels and avoidant coping between young adult offspring of parents with a mental illness and a comparison group not affected by parental mental illness, a one-way MANOVA was conducted using 11 dependent variables (the coping subscales) with group as the between-subjects variable. The analysis revealed a significant overall effect, *F* (11,127) = 2.28, *p* = 0.014, partial η^2^ = 0.17, Wilk’s *Λ* = 0.84.

*Post hoc* univariate ANOVAs were conducted for each dependent variable. A statistically significant difference was found between the two groups on the positive reframing scale, *F* (1,137) = 5.03, *p* = 0.027, partial η^2^ = 0.04, with offspring of parents with a mental illness showing significantly lower scores. Similarly, a significant difference was observed for the behavioral disengagement scale, *F* (1,137) = 4.46, *p* = 0.036, partial η^2^ = 0.03, with these offspring reporting significantly higher scores. No significant differences were found for the other scales.

The results of the multivariate analysis regarding the impact of parental mental illness on the coping strategies of emerging adults are displayed in Table 1.

**Table 1:** Multivariate and univariate analyses of parental mental illness on coping strategies of emerging adults.

Measure	Parent with a mental illness (*n* = 50) Mean (*SE*)	Parent without a mental illness (*n* = 89) Mean (*SE*)	Statistics
COPING STRATEGIES			*F* (11, 127) = 2.28 *p* = 0.014, partial η^2^ = 0.17, Wilk’s *Λ* = 0.84
Active coping	1.65 (0.85)	1.49 (0.69)	*F* (1, 137) = 1.47, *p* = 0.228, partial η^2^ = 0.01
Positive reframing	1.20 (0.89)	1.54 (0.86)	*F* (1, 137) = 5.03, *p* = 0.027, partial η^2^ = 0.04
Acceptance	1.81 (0.76)	1.63 (0.82)	*F* (1, 137) = 1.54, *p* = 0.217, partial η^2^ = 0.01
Humor	1.36 (0.94)	1.49 (0.86)	*F* (1, 137) = 0.67, *p* = 0.413, partial η^2^ = 0.01
Religion	0.53 (0.82)	0.64 (0.87)	*F* (1, 137) = 0.54, *p* = 0.465, partial η^2^ = 0.00
Emotional support	1.54 (0.95)	1.77 (0.90)	*F* (1, 137) = 2.02, *p* = 0.157, partial η^2^ = 0.02
Instrumental support	1.26 (0.78)	1.30 (0.91)	*F* (1, 137) = 0.08, *p* = 0.778, partial η^2^ = 0.00
Denial	0.78 (0.85)	0.58 (0.72)	*F* (1, 137) = 2.10, *p* = 0.150, partial η^2^ = 0.02
Substance use	0.50 (0.83)	0.29 (0.57)	*F* (1, 137) = 3.20, *p* = 0.076, partial η^2^ = 0.02
Behavioral disengagement	0.86 (0.96)	0.57 (0.66)	*F* (1, 137) = 4.46, *p* = 0.036, partial η^2^ = 0.03
Self-blame	1.31 (0.94)	1.22 (0.85)	*F* (1, 137) = 0.32, *p* = 0.571, partial η^2^ = 0.00

Additional analyses were performed to test the potential impact of sex and emerging adult’s mental health status. A two-way MANOVA with sex as a second factor and the 11 subscales of the Brief COPE as dependent variables demonstrated no significant effect of sex, *F* (22,248) = 0.87, *p* = 0.641, partial η^2^ = 0.07, Wilk’s *Λ* = 0.86, and no interaction effect, *F* (11,124) = 1.83, *p* = 0.056, partial η^2^ = 0.14, Wilk’s Λ = 0.86. The effect of the parental mental health status remained significant, *F* (11,124) = 2.07, *p* = 0.028, partial η^2^ = 0.155, Wilk’s Λ = 0.85.

A one-way MANOVA with the grand mean-centered GSI of the Mini-SCL as a covariate indicated that the covariate was significantly related to coping strategies, *F* (11,126) = 9.02, *p* < 0.001, partial η^2^ = 0.44, Wilk’s Λ = 0.56. Subsequent univariate analyses revealed significant effects of mental health status on several subscales: humor, *F* (1,136) = 5.46, *p* = 0.021, partial η^2^ = 0.04; denial, *F* (1,136) = 28.02, *p* < 0.001, partial η^2^ = 0.17; substance use *F* (1,136) = 22.21, *p* < 0.001, partial η^2^ = 0.14; behavioral disengagement, *F* (1,136) = 15.44, *p* < 0.001, partial η^2^ = 0.10; and self-blame, *F* (1,136) = 53.85, *p* < 0.001, partial η^2^ = 0.28. Subsequent correlational analyses demonstrated that all significant subscales were significantly positively correlated with the global severity index, with correlations ranging from *r* = 0.17 (humor) to *r* = 0.52 (self-blame).

### 3.3 Regression analyses

To test possible associations between coping - especially avoidant coping - and mental stress and positive mental health status, two regression analyses were conducted to quantify the variance in symptoms of psychopathology and positive mental health accounted for by different coping strategies. The significant relationships identified in the regression analyses are consistent with the findings from the correlational analyses (see Supplementary Table S4). Table 2 presents the regression equations estimating associations between young adults’ symptoms of psychopathology and coping variables.

**Table 2:** Regression equations estimating associations between coping strategies and symptoms of psychopathology (Mini-SCL GSI).

Coefficients	*b*	95% CI of *b*	*SE*	*ß*	*t*	*p*
	Final *R^2^* = 0.54 *F* (11,38) = 4.12, *p* < 0.001					
Subgroup of young adult children of parents with a mental illness (*n* = 50)						
Constant	0.92	−12.30, 14.14	6.53		0.14	0.889
Active coping	3.12	−1.66, 7.90	2.36	0.20	1.32	0.195
Positive reframing	2.20	−1.85, 6.25	2.00	0.15	1.10	0.279
Acceptance	0.25	−4.69, 5.18	2.44	0.01	0.10	0.920
Humor	−1.79	−5.80, 2.21	2.00	−0.13	−0.91	0.371
Religion	−3.42	−7.60, 0.76	2.06	−0.21	−1.66	0.106
Emotional support	0.12	−4.41, 4.65	2.24	0.01	0.05	0.959
Instrumental support	−0.81	−6.17, 4.56	2.65	−0.05	−0.30	0.762
Denial	1.38	−3.04, 5.81	2.19	0.09	0.63	0.531
Substance use	4.50	0.44, 8.56	2.01	0.28	2.24	0.031
Behavioral disengagement	2.43	−1.27, 6.14	1.83	0.18	1.33	0.192
Self-blame	7.77	3.30, 12.25	2.21	0.55	3.52	0.001
Subgroup of young adult children of parents without a mental illness (*n* = 89)						
	Final *R^2^* = 0.46 *F* (11,77) = 5.87, *p* < 0.001					
Constant	7.53	−0.35, 15.40	3.95		1.90	0.061
Active coping	−1.58	−5.01, 1.89	1.74	−0.10	−0.90	0.369
Positive reframing	−1.24	−3.86, 1.38	1.32	−0.10	−0.94	0.348
Acceptance	0.40	−2.59, 3.39	1.50	0.03	0.27	0.790
Humor	1.08	−1.76, 3.92	1.43	0.08	0.76	0.451
Religion	−1.02	−3.65, 1.62	1.33	−0.08	−0.77	0.446
Emotional support	−2.71	−5.90, 0.49	1.61	−0.22	−1.69	0.096
Instrumental support	1.95	−1.11, 5.00	1.54	0.16	1.27	0.209
Denial	4.03	0.87, 7.19	1.59	0.26	2.54	0.013
Substance use	3.04	−0.65, 6.73	1.85	0.16	1.64	0.105
Behavioral disengagement	1.82	−2.27, 5.92	2.06	0.11	0.89	0.378
Self-blame	4.50	1.90, 7.10	1.31	0.32	3.45	<0.001

For young adult children of parents with a mental illness, the model was significant, *F* (11,38) = 4.12, *R^2^* = 0.54, *p* < 0.001. Within this model, greater use of substance use, *b* = 4.50, *ß* = 0.28, *t* (38) = 2.24, *p* = 0.031, and self-blame, *b* = 7.77, *ß* = 2.21, *t* (38) = 3.52, *p* = 0.001, was associated with more symptoms of psychopathology.

For young adults who did not report a parental mental illness, the model was significant, *F* (11,77) = 5.87, *R^2^* = 0.46, *p* < 0.001. In this model, a greater use of denial, *b* = 4.03, *ß* = 0.26, *t* (77) = 2.54, *p* = 0.013, and self-blame, *b* = 4.50, *ß* = 0.32, *t* (77) = 3.45, *p* < 0.001, was associated with more symptoms.

Table 3 presents the regression equations for young adults’ positive mental health status as a function of their reported coping strategies. For young adults with parents with a mental illness, the model was significant, *F* (11,38) = 5.12, *R^2^* = 0.60, *p* < 0.001, with greater use of substance use, *b* = −0.21, *ß* = −0.27, *t* (38) = −2.30, *p* = 0.027, and self-blame, *b* = −0.28, *ß* = −0.42, *t* (38) = −2.81, *p* = 0.008, being associated with worse mental health status.

**Table 3:** Regression equations estimating associations between coping strategies and positive mental health (PMHS).

Coefficients	*b*	95% CI of *b*	*SE*	*ß*	*t*	*p*
Subgroup of young adult children of parents with a mental illness (*n* = 50)						
	Final *R^2^* = 0.60 *F* (11,38) = 5.12, *p* < 0.001					
Constant	1.79	1.21, 2.38	0.29		6.19	<0.001
Active coping	0.01	−0.20, 0.22	0.11	0.01	0.10	0.921
Positive reframing	−0.08	−0.26, 0.10	0.09	−0.11	−0.86	0.396
Acceptance	0.20	−0.02, 0.42	0.11	0.24	1.85	0.073
Humor	−0.04	−0.21. 0.14	0.09	−0.05	−0.40	0.692
Religion	0.12	−0.07. 0.30	0.09	0.16	1.30	0.202
Emotional support	0.09	−0.11, 0.29	0.10	0.14	0.93	0.356
Instrumental support	−0.03	−0.27, 0.21	0.12	−0.04	−0.27	0.786
Denial	−0.02	−0.22, 0.18	0.10	−0.03	−0.20	0.843
Substance use	−0.21	−0.39, −0.02	0.09	−0.27	−2.30	0.027
Behavioral disengagement	−0.09	−0.26, 0.07	0.08	−0.14	−1.12	0.270
Self-blame	−0.28	−0.47, −0.08	0.10	−0.42	−2.81	0.008
Subgroup of young adult children of parents without a mental illness (*n* = 89)						
	Final *R^2^* = 0.43 *F* (11,77) = 5.29, *p* < 0.001					
Constant	1.90	1.46, 2.34	0.22		8.60	<0.001
Active coping	−0.11	−0.31, 0.08	0.10	−0.13	−1.14	0.260
Positive reframing	0.18	0.03, 0.32	0.07	0.25	2.38	0.020
Acceptance	−0.03	−0.20, 0.14	0.08	−0.04	−0.35	0.728
Humor	0.12	−0.04, 0.28	0.08	0.17	1.49	0.142
Religion	0.08	−0.07, 0.23	0.07	0.11	1.04	0.303
Emotional support	0.15+	−0.03, 0.33	0.09	0.22	1.67	0.099
Instrumental support	−0.05	−0.22, 0.13	0.09	−0.07	−0.54	0.592
Denial	−0.22	−0.40, −0.04	0.09	−0.26	−2.47	0.016
Substance use	−0.21	−0.41, 0.00	0.10	−0.19	−1.99	0.050
Behavioral disengagement	−0.15	−0.38, 0.08	0.12	−0.17	−1.33	0.187
Self-blame	−0.15	−0.29, 0.00	0.07	−0.19	−1.20	0.049

For young adults who did not report a parental mental illness, the model was significant, *F* (11,77) = 5.29, *R^2^* = 0.43, *p* < 0.001. In this model, greater use of positive reframing, *b* = 0.18, *ß* = 0.25, *t* (77) = 2.38, *p* = 0.020, was associated with better positive mental health, while greater use of denial, *b* = −0.22, *ß* = −0.26, *t* (77) = −2.47, *p* = 0.016 and self-blame, *b* = −0.15, *ß* = −0.19, *t* (77) = −2.00, *p* = 0.049, was associated with worse mental health status.

## 4 Discussion

The present study aimed to advance understanding of coping among emerging adults at elevated risk, with the goal of identifying specific coping strategies as risk or protective factors. Specifically, we examined whether emerging adult children of parents with a mental illness would use support seeking and avoidant coping strategies more frequently than the comparison group (Goodday et al., 2019; Mitchell and Abraham, 2018). Partially consistent with this hypothesis, the groups differed significantly on two subscales: participants with parental mental illness reported lower scores on positive reframing and higher scores on behavioral disengagement.

These results are inconsistent with findings by Goodday et al. (2019), who reported significantly greater use of support seeking in an elevated-risk sample. However, Goodday et al. examined not only emerging adults but also children and adolescents, age groups for whom research indicates greater reliance on support seeking strategies (Kahl and Jungbauer, 2014; Maybery et al., 2005; Valdez et al., 2019).

Although no significant between-group differences emerged in the use of support seeking strategies, both emotional and instrumental support were reported at high levels in both groups. This pattern aligns with prior qualitative research documenting extensive support seeking among offspring exposed to parental mental illness (Sawitzki et al., 2025). Nevertheless, the absence of mean differences does not preclude group-specific patterns in how support is mobilized. If parental support is limited – or if participants seek to avoid burdening a parent with mental illness – emerging adults may substitute other sources, such as friends, siblings, instructors, or health professionals. They may also rely more on anonymous online resources (Matar et al., 2018) and vary their sources depending on the domain of need. Moreover, the present findings speak to the quantity of support reported rather than its quality; responsiveness, perceived helpfulness, and trustworthiness may differ between groups.

Among avoidant coping subscales, only the subscale behavioral disengagement differed significantly, indicating higher use among emerging adults with a parent with mental illness. Behavioral disengagement is the tendency to reduce “one’s effort to deal with the stressor, even giving up the attempt to attain goals with which the stressor is interfering” (Carver et al., 1989, p. 269). In contexts characterized by low controllability, such as parental mental illness, behavioral disengagement could be construed as an adaptive response (Forsythe and Compas, 1987) and therefore may be used more frequently. In addition, most participants in the present sample reported that their parents had a mood disorder, raising the possibility that parents may have modeled behavioral disengagement as a coping strategy (Goodman, 2020). Nevertheless, mean scores for behavioral disengagement were low in both groups.

Positive reframing was reported significantly less often by emerging adult children of parents with a mental illness than by the comparison group. Family contexts characterized by instability and unpredictability may foster learned helplessness, rendering positive reframing less accessible and reducing the cognitive and emotional resources available for reframing (Seligman, 1972). Moreover, because most participants reported that their parents had a mood disorder, parents may have modeled less frequent use of positive reframing (Goodman, 2020).

Although no significant between-group differences emerged on the acceptance subscale, it was the most frequently endorsed coping strategy among emerging adults with a parent with mental illness. This pattern is consistent with prior research (Sawitzki et al., 2025) and suggests some degree of resilience within this elevated-risk group.

Concerning the associations between coping and mental health, greater use of substance use, and self-blame was associated with higher psychopathology symptom severity and lower positive mental health. By contrast, denial and behavioral disengagement were not significantly associated with the health outcomes. This pattern contrasts with Goodday et al. (2019), who found a significant positive association between avoidant coping and risk of developing an affective disorder. A key difference is that Goodday et al. examined longitudinal associations with onset and recurrence of mood episodes, whereas the present analyses were cross-sectional. The deleterious effects of avoidant coping strategies may emerge primarily over time, as individuals encounter fewer opportunities to experience self-efficacy, anxiety is maintained, and problems remain unaddressed, cumulatively increasing burden over the long term (Skinner and Zimmer-Gembeck, 2016).

Consistent with prior research, self-blame was positively associated with psychopathology symptoms and inversely associated with positive mental health in both groups. Studies have repeatedly linked self-blame to poorer mental health among offspring of parents with mental illness (Fear et al., 2009; van der Sanden et al., 2016) and in general-population samples without parental mental illness (Buizza et al., 2022; Jannati et al., 2020). Likewise, greater substance use as coping strategy was associated with poorer mental health among emerging adult children of parents with a mental illness.

### 4.1 Limitations and future research

Several limitations of the present study should be noted. The cross-sectional design precludes causal inference and does not establish directionality; coping may influence psychopathology, and symptoms may also shape coping (Richardson et al., 2021). Moreover, parental mental disorders were reported by offspring rather than independently verified through clinical assessment, potentially introducing recall bias (over- or underestimating the parental psychopathology). Our analysis did not differentiate between specific types of parental mental illness, which might have masked disorder-specific patterns in the findings. Additionally, the generalizability of the current study’s findings is limited due to the non-representative nature of the sample, which consisted primarily of German, female, and academically affiliated participants. Moreover, with a larger sample size, the study might have had greater statistical power to detect additional effects. Future research should employ longitudinal designs; strive to include more male and gender-diverse participants as well as from diverse sociodemographic, cultural, and family backgrounds; and examine both the sources and perceived quality of social support. To minimize potential recall-bias, objective clinical assessments of parental mental illness should be implemented. Disorder-specific analyses may reveal distinct risk profiles associated with different mental health conditions. Additionally, replication studies with larger samples are needed to validate our current findings and to enhance statistical power for detecting potential effects. Investigating coping profiles (Paquette et al., 2025), or coping flexibility (Kato, 2012) – rather than isolated strategies – may also be promising.

### 4.2 Conclusion and implications

In sum, emerging adults with a parent with mental illness reported significantly higher behavioral disengagement and significantly lower positive reframing than the comparison group. Greater self-blame and substance use were associated with higher distress and lower positive mental health. These findings refine understanding of coping-related risk and protective factors in the transgenerational transmission of psychopathology and identify coping as a promising target for prevention and intervention. For example, findings of an evaluation of the online program mi.spot (Maybery et al., 2022) align with the present study’s implications: increases in positive reframing and decreases in self-blame may benefit emerging adults with a parent with mental illness.

## Data Availability

The datasets presented in this study can be found in online repositories. The names of the repository/repositories and accession number(s) can be found PsychArchives, https://doi.org/10.23668/psycharchives.16064.
