# Supplementary material for: Coping strategies among emerging adult children of parents with a mental illness
Source: Front Psychol. 2026 Apr 15;17:1760621. doi: 10.3389/fpsyg.2026.1760621 (PMC13124927; doi:10.3389/fpsyg.2026.1760621)
Supplement: Supplementary file 1 [file Table_1.docx]

Supplementary Material

# Supplementary Data

Table S1: Demographic Characteristics of Participants With and Without a Parent With a Mental Illness.

| Demographic Variable | Participants with parents with a mental illness  (*n* = 50) | Participants with parents without a mental illness  (*n* = 89) | Significance Test |
| --- | --- | --- | --- |
|  | **Mean (*SE*)** | **Mean (*SE*)** |  |
| Age | 22.40 (2.72) | 22.64 (2.99) | *t*(137) = -.47, *p =*.639,  *d*= 2.90, 95% CI for Cohen's *d* [-.43, .26] |
| Subjective social status | 5.74 (1.77) | 6.20 (1.65) | *t*(137) = -.16, *p =*.124, *d*= 1.69, 95% CI for Cohen's *d*  [-.62, .08] |
|  | **Frequency (%)** | **Frequency (%)** |  |
| Gender | Female: 46 (92.0)  Male: 2 (4.0)  Diverse: 2 (4.0) | Female: 72 (80.9)  Male: 17 (19.1)  Diverse: 0 (0.0) | *p=* .005 |
| Educational attainment | Lower than A-levels:  0 (0,0)  A-levels: 44 (88.0)  Bachelor: 4 (8.0)  Master: 2 (4.0)  Doctor: 0 (0.0) | Lower than A-levels:  7 (7.9)  A-levels: 67 (75.3)  Bachelor: 11 (12.4)  Master: 3 (3.4)  Doctor: 1 (1.1) | *p=* .165 |
| Employment status | School pupil: 1 (2.0)  Student: 38 (76.0)  Employee: 9 (18.0)  Self-employed: 1 (2.0)  Unemployed: 1 (2.0) | School pupil: 5 (5.6)  Student: 59 (66.3)  Employee: 23 (25.8)  Self-employed: 2 (2.2)  Unemployed: 0 (0.0) | *p=* .418 |
| Religion | Christianity: 20 (40.0)  Islam: 1 (2.0)  Buddhism: 1 (2.0)  Hinduism: 0 (0.0)  None: 26 (52.0) | Christianity: 49 (55.1)  Islam: 5 (5.6)  Buddhism: 0 (0.0)  Hinduism: 1 (1.1)  None: 31 (43.8) | *p=* .088 |
| Nationality | German: 49 (98.0)  Other (or dual): 1 (2.0) | German: 84 (94.4)  Other (or dual):  5 (5.6) | *p=* .419 |
| Migration background | Yes: 13 (26.00)  No: 37 (74.00) | Yes: 19 (21.30)  No: 70 (78.70) | X² (1) = .39,  *p*= .532, φ = .05 |

*Note: t-test was used for age and socioeconomic status, the Chi-Square-Test for migration background, and Fisher’s Exact Test for all other variables.*

Table S2: Participant-Reported Characteristics of Their Parents‘ Mental Illness and Background Information.

| Characteristics |  | |
| --- | --- | --- |
|  | **Frequency (percentage)** | |
| Gender of parent with mental illness | Mother: 26 (52.0)  Father: 14 (28.0)  Both: 10 (20.0) | |
|  | | |
| Diagnosis (according to the DSM-5 diagnostic criteria) | **Mother** | **Father** |
| Schizophrenia spectrum and other psychotic disorders | 1 (3.03) | 1 (4.55) |
| Bipolar and related disorders | 4 (12.12) | 0 (0.00) |
| Depressive disorders | 13 (39.39) | 11 (50.00) |
| Anxiety disorders | 1 (3.03) | 0 (0.00) |
| Obsessive-compulsive disorder and related disorders | 1 (3.03) | 0 (0.00) |
| Trauma and stress-related disorders | 0 (0.00) | 1 (4.55) |
| Disorders related to psychotropic substances and addictive behaviors | 0 (0.00) | 4 (18.18) |
| Neurocognitive disorders (NCD) | 0 (0.00) | 1 (4.55) |
| Personality disorders | 5 (15.15) | 2 (9.09) |
| Comorbid, multiple disorders | 8 (24.24) | 2 (9.09) |
| Frequency of contact with parent with mental illness | Daily: 7 (14.00)  Once a week: 13 (26.00)  Once a month: 6 (12.00)  Less than once a month: 2 (4.00)  No contact: 6 (12.00)  (Rest is missing) | |
|  | **Mean (*SE*)** | |
| Duration of living together (in years) | 17.89 (4.67) | |
| Age when illness became conscious | 11.59 (4.28) | |

Table S3: Mental Health Status of Emerging Adult Children.

| Variable measuring health status | Offspring of a parent with mental illness (*n*= 50) | Offspring of a parent without mental illness (*n* = 89) | Significance test |
| --- | --- | --- | --- |
|  | Mean (*SE*) | Mean (*SE*) |  |
| Mini-SCL: Depression | 7.62 (6.35) | 4.70 (4.74) | *U*=1576.50,  *Z*= -2.86, *p =*.004 |
| Mini-SCL: Anxiety | 7.66 (5.60) | 4.62 (4.64) | *U*=1466.50,  *Z*= -3.34, *p*< .001 |
| Mini-SCL: Somatization | 4.38 (4.37) | 3.09 (3.50) | *U*=1852.50,  *Z*= -1.65, *p =*.099 |
| Mini-SCL: GSI | 19.66 (13.22) | 12.40 (11.07) | *U*=1455.00,  *Z*= -3.43, *p <*.001 |
| PMHS | 1.64 (.62) | 1.94 (.61) | *U*=1575.00,  *Z*= -2.86, *p*= .004 |

*Note: As requirements for MANOVA were violated, Mann Whitney U-Tests were calculated.*

Table S4: Correlational Analyses

| **Subgroup of young adult children of parents with a mental illness (n=50)** | | | **Subgroup of young adult children of parents without a mental illness (n=89)** | |
| --- | --- | --- | --- | --- |
|  | Mini-SCL: GSI | PMHS | Mini-SCL: GSI | PMHS |
| Mini-SCL: GSI | - |  | - |  |
| PMHS | -.748*** | - | -.584*** | - |
| Active Coping | .077 | .184 | -.239* | .146 |
| Positive Reframing | -.011 | .164 | -.236* | .365*** |
| Acceptance | -.195 | .435** | .076 | .026 |
| Humor | .256 | -.396** | .154 | .069 |
| Religion | -.221 | .295* | -.031 | .104 |
| Emotional Support | -.027 | .232 | -.200 | .268* |
| Instrumental Support | -.066 | .226 | -.120 | .164 |
| Denial | .409** | -.307* | .417*** | -.390*** |
| Substance Use | .386** | -.419** | .367*** | -.325** |
| Behavioral Disengagement | .215 | -.232 | .423*** | -.338** |
| Self-Blame | .598*** | -.628*** | .481*** | -.353*** |

*Note: *p < .05. **p < .01. ***p < .001.*

# Supplementary information: Prompt used in LibreChat

Bitte überprüfe den folgenden Text auf Rechtschreibung, Zeichensetzung und grammatikalische Korrektheit. Achte außerdem auf Lesbarkeit, Kohärenz, Struktur und sprachlichen Fluss. Stelle sicher, dass der Ton höflich, formell und akademisch bleibt. Bewahre den ursprünglichen Schreibstil, verfeinere ihn aber dort, wo es nötig ist, um die Klarheit und Professionalität des Textes zu erhöhen. Vermeide umgangssprachliche Ausdrücke und bevorzuge einen formellen, wissenschaftlich geprägten Stil. Gib direktes, präzises und hilfreiches Feedback, das mir dabei hilft, den Text effizient zu verbessern – insbesondere für den Einsatz im beruflichen oder akademischen Kontext.
